# Supplementary material for: Patterns of Intron Gain and Loss in Fungi
Source: PLoS Biol. 2004 Nov 30;2(12):e422. doi: 10.1371/journal.pbio.0020422 (PMC532390; doi:10.1371/journal.pbio.0020422)
Supplement: Table S1 — Also available at http://genes.mit.edu/NielsenEtAl/. (4.3 MB ZIP). [file pbio.0020422.st001.zip › NielsenEtAl/html/250.html]

AN7108.1.NCU08827.1.MG06149.1.FG09644.1


```
 CLUSTAL W (1.82) Multiple Sequence Alignments - Introns Inserted


Sequence 1: NCU08827.1	341 aa
Sequence 2: MG06149.1	311 aa
Sequence 3: FG09644.1	291 aa
Sequence 4: AN7108.1	377 aa
Alignment Length: 428 aa
Number Identitical Residues: 83 aa
Alignment Score (without introns) 4946


MG06149.1 	------------MAAVISSAT---------PDAPLQSRIVDDKSPICIPFILERLSVWRK
NCU08827.1	MMDTIDSISSQPTAAATSTSTSTSSSSGVNPTPSPQSRIIDDKSPLCIPFILSKIAEYNA
FG09644.1 	----------------------------------MAPHFVDDKTEICLPFILAQL----K
AN7108.1  	-----------------------------------MPEIFDDKSQHCIPFLLQRLKAHQA
          	                                    ..:.***:  *:**:* ::     

MG06149.1 	ARD-ENDRRP-FFIGLNGVQGVGKTTL~VRALAETLQQREM-LETIVVSIDDFYLGHQTQ
NCU08827.1	LHANEANPRP-FIIGLNGVQGVGKTTL~VKALAETLQEREG-LNTLVVSIDDFYLTHEEQ
FG09644.1 	LHREESPDRP-FVIGLNGIQGAGKSTL~VKSLSKALESQN--VPTLVCSIDEFYLTRQDQ
AN7108.1  	RHSNDPANTPPFFLGLNGVQGAGKTVL0VSTLNSILRSPPYSLAVVTLSLDDLYLTHEDQ
          	 : .:    *.*.:****:**.**:.* * :* . *..   :: .:. *:*::** :: *

MG06149.1 	LALAESQPDNSLVQYRGEPG1THDIELMQSFFTAVINGQPAKIPQYDKGAYSGLGDRAPE
NCU08827.1	LKLAEENSDNALVQYRGEPG1THDTPLLLSFLSSIISSQPTHLPLYDKSAHHGLGDRLPA
FG09644.1 	VALAESDPENALIQHRGEPG1THDLPLAKAFFEAVLKGEPTKLPKYDKAIKGGKGDRLPE
AN7108.1  	VSLAKSNPTNPLLQHRGQPG~THDLPLAQSVFSALRAGRPTAIPQYDKSAFSGQGDRVPM
          	: **:.:. *.*:*:**:** ***  *  :.: ::  ..*: :* ***.   * *** * 

MG06149.1 	STWPSIN-----GLDQRPVEVVLVEGWCVGFRALEPEAVVEKWRSPDT---------RTL
NCU08827.1	STFPAINDPNLSAPQHRTIRVLLLEGWLTGFRSLPPGTISTKYLDLKSY--------KTL
FG09644.1 	SEWTLVN-----QPGQPKIQAIILEGWCVGFRPLTREGVEARLNMPNR----------TL
AN7108.1  	EQWETVN-----VEGQEQIKVLIFEGWSVGFRAWDDDILKQKWEDAVKRKEQDKEYRGRL
          	. :  :*        :  :..::.*** .***.     :  :       ......    *

MG06149.1 	RNHRTEHLLFVNEKLGAYDVLTN~QLDALVHIDAQKLEYVYDWRQEQEAQLRKERG---T
NCU08827.1	SHHKLEHLLFINERLKEYEPAWD~QFDAFIHIDAQDLEWVYEWRLEQEEQLRREKG---T
FG09644.1 	KQHKLEHLLFINEKLSEYNTLTD~SFDSFIQIDAEDLGYVYGWRLEQEDHLREERGDPEA
AN7108.1  	GHVKFEDVRKVNEALRGYDVLTD2--------DAQDNHFVYEWRQEQERTLRATKG---I
          	 : : *.:  :** *  *:   :         **:.  :** ** ***  **  :*    

MG06149.1 	GMSDEQVVRFVDAY~YPAYELYSDGVRAGVLLP--ERPGCQ-------LT~LI~VD----
NCU08827.1	GMSAEMVRKFVDGY~FPAYELYEDGVRQGVFKGDEEKRGRQ-------LR~MV~VG----
FG09644.1 	GMCSQEVVKFVDGY~YPAYELYTDRLRKGVLAN---RPGRQ-------LR~MV0N-----
AN7108.1  	GMTEEQVNRFVDGS1RLSAQEHSDRFRIRLRHLRRRRAGRGDNFDWLSIK0II~LTTMDE
          	**  : * :***.    : : : * .*  :      : *  .. .  ::  ::   : ..

MG06149.1 	---RHRRVVEHFVL----------------------------------------------
NCU08827.1	---RDRGVVEHMVI----------------------------------------------
FG09644.1 	----HHIPLQTA------------------------------------------------
AN7108.1  	TYFIHRRSPKNMHEEIVPSQPDSLRSMQLREEPQIWKRRMQCRRTRLPQMTAFNDIGVNV
          	:   .:   :    .  .:...:  : .  ....  .   ..  :  .. :: .. . . 

MG06149.1 	--~------------
NCU08827.1	--~------------
FG09644.1 	--~------------
AN7108.1  	DK0KDYNLPLIMERG
          	.. .. . .   . .
```
